# The use of psychiatric services by young adults who came to Sweden as teenage refugees: a national cohort study

**DOI:** 10.1017/S2045796016000445

**Published:** 2016-06-29

**Authors:** H. Manhica, Y. Almquist, M. Rostila, A. Hjern

**Affiliations:** 1 CHESS Centre for Health Equity Studies, Centre for Health Equity Studies (CHESS), Stockholm University/Karolinska, Sveaplan, Sveavägen 160, Floor 5, Stockholm, Stockholm, Sweden; 2 Centre for Health Equity Studies (CHESS), Stockholm University/Karolinska Institute, Stockholm, Sweden; 3 Department of Medicine, Karolinska Institute and Centre for Health Equity Studies, Stockholm, Sweden

**Keywords:** Adolescents, discrimination, mental health, primary care, psychiatric services

## Abstract

**Aims.:**

To investigate the patterns of use of different forms of psychiatric care in refugees who settled in Sweden as teenagers.

**Method.:**

Cox proportional hazards models were used to estimate the use of different forms of psychiatric care from 2009 to 2012 in a population of 35 457 refugees, aged from 20 to 36, who had settled in Sweden as teenagers between 1989 and 2004. These findings were compared with 1.26 million peers from the same birth cohorts in the general Swedish population.

**Results.:**

Unaccompanied and accompanied refugees were more likely to experience compulsory admission to a psychiatric hospital compared with the native Swedish population, with hazard ratios (HRs) of 2.76 (1.86–4.10) and 1.89 (1.53–2.34), respectively, as well as psychiatric inpatient care, with HRs of 1.62 (1.34–1.94) and 1.37 (1.25–1.50). Outpatient care visits by the young refugees were similar to the native Swedish population. The longer the refugees had residency in Sweden, the more they used outpatient psychiatric care. Refugees born in the Horn of Africa and Iran were most likely to undergo compulsory admission, with HRs of 3.98 (2.12–7.46) and 3.07 (1.52–6.19), respectively. They were also the groups who were most likely to receive inpatient care, with HRs of 1.55 (1.17–2.06) and 1.84 (1.37–2.47), respectively. Our results also indicated that the use of psychiatric care services increased with the level of education in the refugee population, while the opposite was true for the native Swedish population. In fact, the risks of compulsory admissions were particularly higher among refugees who had received a secondary education, compared with native Swedish residents, with HRs of 4.72 (3.06–7.29) for unaccompanied refugees and 2.04 (1.51–2.73) for accompanied refugees.

**Conclusions.:**

Young refugees received more psychiatric inpatient care than the native Swedish population, with the highest rates seen in refugees who were not accompanied by their parents. The discrepancy between the use of inpatient and outpatient care by young refugees suggests that there are barriers to outpatient care, but we did note that living in Sweden longer increased the use of outpatient services. Further research is needed to clarify the role that education levels among Sweden's refugee populations have on their mental health and health-seeking behaviour.

## Background

Refugees and their families have dominated immigration to Sweden since the mid-1970s. Epidemiological studies of young refugees have shown high rates of psychological distress and behavioural problems during the first years of resettlement (Davidson *et al.* 2004; Bean *et al.* 2007*b*; Kirmayer *et al.* 2011; Fazel *et al.* 2012). Scandinavian follow-up studies on the mental health of child refugees after resettlement have shown a high prevalence of psychological distress on arrival, with considerable improvement over time (Hjern & Angel, 2000; Montgomery, 2010).

Over the last decade, Europe, and in particular Sweden, has received increasing numbers of unaccompanied refugee children, mostly male teenagers from the Horn of Africa, Afghanistan, Iraq and Syria (Hjern, 2012; Eide & Hjern, 2013; Migrationsverket, 2015). High levels of depressive symptoms have been reported by different European studies on this population (Bean *et al.* 2007*a*; Derluyn *et al.* 2009; Seglem *et al.* 2011; Ramel *et al.* 2015), suggesting that unaccompanied refugees are at a particularly high risk of mental health problems, especially introverted symptoms.

The high rate of poor mental health among newly settled refugee children and young people has been explained by multiple stress factors related to their refugee status. These include experiences of war, the loss of family members, separation from families and friends, the asylum process and living arrangements (Howard & Hodes, 2000; Kirmayer *et al.* 2011; Hjern, 2012). Socio-economic factors and living on the margins of society have also been associated with increased rates of mental health problems and suicide attempts (Jablonska *et al.* 2009), psychotic disorders (Hjern *et al.* 2004) and illicit drug abuse (Hjern, 2004). Discrimination due to physical appearance has been suggested as a chronic stressor that, in itself, can increase the risk of psychiatric disorders (Scheppers *et al.* 2006; Bean *et al.* 2007*a*; Hjern, 2012).

The aim of this study was to investigate the patterns of different forms of psychiatric care usage by young refugees, a minimum of 4 years after they had been granted residency in Sweden as teenagers.

Firstly, we were keen to discover whether there were differences between accompanied and unaccompanied refugees compared with the native Swedish population. We hypothesised that risk factors for mental health problems would be severely compounded among unaccompanied refugees, as they lacked the social and emotional support they needed from their families to cope with the stressors due to traumatic experiences, both during migration and in the post-migration period. Therefore, we expected that unaccompanied refugees would make more use of mental healthcare than accompanied refugees and native Swedish residents. Secondly, we hypothesised that the refugees’ access to, and use of, psychiatric care services would be influenced by socio-cultural factors that depended on their country of origin and the socio-economic conditions offered by the country they were migrating to. Therefore, this study investigated whether patterns differed between refugees from different backgrounds and to what extent socio-economic factors explained differences in the use of psychiatric care between the refugee population and the general Swedish population. Finally, we also wanted to investigate to what extent length of residence was associated with use of psychiatric care services. We expected to see a time-dependent pattern and we hypothesised that the use of psychiatric care would increase with time spent living in Sweden, as newly arrived refugees became more familiar with accessing mental healthcare services.

## Methods

Sweden has a long tradition of maintaining national registers that provide high-quality data on health and socio-economic indicators. These are protected by special legislation, which makes it possible to collect certain information without the personal consent of individuals (Rosén, 2002). These registers can be linked to each other using the unique personal identification number that follows Swedish residents from birth or immigration to death. In this register-based study, we linked immigration and socio-demographic data from registers held by Statistics Sweden to health data held by the Swedish Migration Agency of Health and Welfare. The study was approved by the Regional Ethics Committee in Stockholm before any records were linked.

### Study population

The study population comprised individuals who were born between 1972 and 1988 and were, according to the Register of the Total Population (RTP), alive and living in Sweden on 31 December 2004 and 31 December 2008. This data source enabled us to identify 35 457 individuals who were between the ages of 20 and 36 in 2008 and were between 13 and 19 years of age when they received permanent residency in Sweden as refugees or because they were related to a refugee.

The information on refugees was acquired from STATIV, a longitudinal database for integration studies, which is held by Statistics Sweden and based on data from the Swedish Migration Agency. Our definition of a refugee was an individual who complied with Article 1 of the 1951 Geneva Convention Relating to the Status of Refugees and the 1967 Protocol, as well as those granted residence permits in Sweden on humanitarian grounds. We compared these 35 457 refugees with the entire population from the same birth cohorts who were born in Sweden and whose parents were also born in Sweden. When we excluded adopted children and those born to parents who were foreign-born, this provided 1 255 782 individuals.

The study population was followed up in the National Patient Discharge Register for specialist psychiatric care from 1 January 2009 to 31 December 2012. The study design ensured that everyone in the teenage refugee population had lived in Sweden for at least 4 years, so they had had their first experience of the Swedish medical care system, as suggested by a previous study (Brendler-Lindqvist *et al.* 2014).

### Main predictors

Information on the refugees’ country of birth was obtained from the RTP and categorised into: former Yugoslavian republics (*n* = 10 477), Horn of Africa (*n* = 4093), Iran (*n* = 2725), Iraq (*n* = 7549) and others (*n* = 10 613), which included South Asia, Latin America, Africa and the Middle East. The Horn of Africa included Somalia, Eritrea and Ethiopia. The teenage refugees were categorised as accompanied (*n* = 29 081) if they had obtained residency because they were related to a family member who was a refugee, according to STATIV, or had at least one parent in the Multi-Generation Register who had received residency in Sweden the same year or before the young refugee, according to the RTP. Young refugees who did not fulfil either of these two criteria were categorised as unaccompanied (*n* = 6376). Length of residence was dichotomised as less than, or more than, 10 years in 2008, and was based on information from the RTP on the year and date a residence permit was issued.

### Outcome

We created three outcomes for the use of hospital affiliated psychiatric care services – compulsory care, inpatient care and outpatient care – according to the Swedish national inpatient and outpatient registers held by the Swedish National Board of Health and Welfare. Use of psychiatric care services was indicated by at least one entry in the respective register between 2009 and 2012, with a main diagnosis of mental and behavioural disorders according to the psychiatry chapter of the tenth edition of the World Health Organization International Classification of Disorders (ICD-10). We excluded diagnoses associated with substance abuse (F10–F19), since these disorders are treated by a separate branch of the Swedish psychiatric care system and will be discussed in a future paper. We categorised the remaining diagnoses into: schizophrenia and other psychotic disorders (F20–F29), affective disorders and depression (F30–F39), post-traumatic stress disorder (F43.1), neurotic and somatoform disorders (F40–43.0, F43.2–F49), behavioural, mental health and physiological disorders (F00–F09, F70–F79 and F80–F89) and emotional disorders (F50–F59, F60–F69 and F90–F99).

We chose 2009 as the first year of this study, because this was the first year that a variable indicating compulsory psychiatric care was included in the inpatient register. The quality of these registers is regularly monitored and the validity has been found to be high for most diagnoses (Ludvigsson *et al.* 2011).

### Covariates

The socio-demographic covariates of age, gender, disposable income, education and domicile were retrieved from the 2008 Longitudinal Integration Database for Health Insurance and Labour Market Studies. Education was split into three categories based on the highest attained education: nine years or less in primary education, 10–12 years in primary and secondary education and 13 years or more in primary, secondary and post-secondary education. The educational level was coded as missing if the information was not recorded. Domicile was split into three categories and defined by the place of residence in 2008: a big city referred to the metropolitan areas of Sweden's three largest cities, Stockholm, Gothenburg and Malmo, a town covered other predominately urban communities and rural covered the remainder.

### Statistical analysis

The analyses were based on person-time in the study, measured from 1 January 2009 to death, the first recorded hospital admission to specialised psychiatric care or the end of the follow-up period on 31 December 2012. Cox proportional hazards models were estimated to compare the use of the three outcome variables of use of hospital affiliated psychiatric care provision – inpatient, outpatient and compulsory care services – using data from the Swedish national inpatient and outpatient registers. We applied the method developed by  (Weitoft *et al.* 1999) in order to minimise any bias caused by unrecorded migration in our study population. For example, a year without any information on household income from labour or other benefits was considered to be an indicator of emigration.

Firstly, we analysed the three outcomes of use of hospital affiliated psychiatric care services between unaccompanied and accompanied refugees with the general Swedish population as the reference category. Each longitudinal outcome variable was analysed with adjustments for gender, age and domicile. Furthermore, we performed additional analyses based on how long the subjects had lived in Sweden, based on up to 10 years and more than 10 years. We used the general Swedish population as the reference category, using the same adjustment procedure presented above.

We also performed an additional analysis of the effect of country of birth on the three outcome variables. Refugees from the former Yugoslavian republics, who were the only European refugees, were used as the reference group. Each regression model was adjusted for refugee category – accompanied or unaccompanied – together with length of stay, age, gender and domicile.

Finally, we carried out interaction analyses of both education and gender for the two categories of refugees in relation to the outcomes. We found no, or minimal, interaction effects for gender, but significant interaction effects for all three outcomes for the two refugee categories. As a result, the analysis of education was stratified.

## Results

The socio-demographic characteristics of the study population are presented in Table 1. The teenage refugees were more like to be male than female, particularly among the unaccompanied refugees. The majority of the refugee populations lived in the metropolitan areas of Sweden's three largest cities, Stockholm, Gothenburg and Malmo. The youngest refugee group was from Iraq, with a mean age of 26 years in January 2009. There were considerable country differences in terms of the level of education: 40% of both the accompanied and unaccompanied refugees from Iran had a post-secondary degree, while lower levels of education were found among refugees from the Horn of Africa and Iraq. Unaccompanied refugees had a slightly lower educational level than accompanied refugees. With regard to the duration of formal residency in Sweden, refugees from the former Yugoslavian republics had the shortest mean duration of residency, while the refugees from Iran had the longest.

**Table 1.** Socio-economic indicators of the study population

Variables	Sweden	Former Yugoslavia		Others		Horn of Africa		Iraq		Iran	
		*a*	*b*	*a*	*b*	*a*	*b*	*a*	*b*	*a*	*b*
*N*	*1.255.782*	*8.993*	*1.484*	*9.335*	*1.278*	*2.232*	*1.861*	*6.393*	*1.156*	*2.128*	*597*
	*%*	*%*	*%*	*%*	*%*	*%*	*%*	*%*	*%*	*%*	*%*
Male											
Gender	51.5	52.2	53.7	52.6	64.7	55.7	60.1	54.1	73.3	55.2	74.5
Mean age											
Age	27.8	30.9	26.9	29.5	25.5	31.2	27.2	27.6	23.7	32.8	28.9
Completed education											
Primary	10	22.3	23.3	33.6	35.7	47.7	41.1	42.5	51.2	20.6	15.7
Secondary	50.7	51.1	54.1	47.9	50.2	42.6	46.6	42.6	37.9	39.5	43.6
Post-sec	39.3	26.6	22.6	18.5	14.1	9.7	12.3	14.9	10.9	39.9	40.7
Length of formal residency in years											
≤10	–	19.8	11.3	42.1	39.6	36.1	17.7	60.2	55.1	29.2	10.9
≥11	–	80.2	88.7	57.9	60.4	63.9	82.6	39.8	44.9	70.8	89.1
Domicile											
Big city	42.7	48.6	51.1	60.2	55.4	70.8	75.6	55.3	60.2	67.4	72.8
Town	43.7	45.7	42.6	35.5	40.1	26.1	22.8	41.5	37.7	30.2	24.3
Rural	13.6	5.7	6.3	4.3	4.5	23.1	1.5	3.2	2.1	3.4	2.9
Income in quintiles											
1	16.3	30.9	23.7	52.7	47.1	54.7	42.3	73.5	49.3	40.5	27.1
2	19.7	23.2	25.4	19.9	22.2	18.3	22.3	12.9	22.2	20.1	16.4
3	20.7	20.5	21.8	12.2	12.9	10.2	12.3	6.8	11.7	14.4	17.4
4	21.8	16.8	16.5	9.5	10.3	10.2	12.9	4.4	9.9	12.1	16.6
5	21.7	8.6	12.7	5.7	7.5	6.6	10.2	2.4	6.9	12.9	22.5

Sweden refers to Native Swedish.

Other countries include South Asia, Latin America, Africa and the Middle East.

a, Accompanied.

b, Unaccompanied.

Table 2 presents the main psychiatric diagnoses for psychiatric patients at discharge from hospital care. A higher proportion of refugees were diagnosed with a psychotic diagnosis in all three forms of care than the general Swedish population, with a particularly high difference between the two groups when it came to compulsory care. Affective disorders were diagnosed in a similar proportion of refugees receiving outpatient care, and in the general population, but in a higher proportion of the general population receiving inpatient care. Post-traumatic stress disorder was diagnosed in a higher percentage of refugees receiving inpatient care.

**Table 2.** Main diagnosis for psychiatric patients at discharge from hospital

Mental and behavioural disorders (F00–F99)	Compulsory care (%)			Inpatient care (%)			Outpatient care (%)		
	Swedish	Unaccompanied	Accompanied	Swedish	Unaccompanied	Accompanied	Swedish	Unaccompanied	Accompanied
Schizophrenia and other psychotic disorders (F20–F29)	27.7	80	59.5	13.1	36	30.4	3.8	15.1	12.6
Affective disorders and depression (F30–F39)	27.3	6.7	17.6	34.1	16.6	24.8	30.1	27.6	26.8
PSTD (F43.1)	9.6	0	12.2	14.5	21.9	21.2	9.5	22.3	19.2
Neurotic and somatoform disorders (F40–F43, F43.2–F49)	8.2	0	6.7	18.5	15.8	12.2	31.2	24.3	27.6
Behavioural, mental and physiological disorders (F00–F09, F70–F79 and F80–F89)	8.6	0	1.3	4.5	0.9	3.5	5.7	2.8	3.6
Emotional disorders (F50–F59, F60–F69 and F90–F99)	18.6	13.3	2.7	15.3	8.8	7.9	19.7	7.9	10.2

Estimated rates of the use of compulsory, inpatient and outpatient psychiatric care services by country of origin suggested that the use of outpatient care services were higher among unaccompanied than accompanied refugees, with the highest rate found among refugees from Iran. On the other hand, the highest rates of compulsory referrals were found among refugees from Iran and the Horn of Africa (see Supplementary Table A). Cox regression analyses indicated that unaccompanied and accompanied refugees were more likely to be admitted to a psychiatric hospital than the general Swedish population, with adjustments for the demographic covariates of gender, age and domicile. The results also suggested that the risks were more pronounced for compulsory psychiatric care, followed by inpatient care services (see Supplementary Table B). Table 3 shows Cox regression models for refugee specific risk factors in the refugee population. The highest risks of compulsory detention were found in refugees from the Horn of Africa, with a hazard ratio (HR) of 3.98 (2.12–7.46), and Iran, with an HR of 3.07 (1.52–6.19). The highest levels of inpatient care were also found in refugees from the Horn of Africa, with an HR of 1.55 (1.17–2.06), and Iran, with an HR of 1.84 (1.37–2.47). However, refugees from the Horn of Africa had the lowest risks of using outpatient care, compared with the reference population of refugees from the former Yugoslavian republics. The highest risks of using outpatient care were found among Iranian refugees. Living in Sweden for longer than 10 years increased the use of outpatient care.

**Table 3.** Cox regression models for first hospital admission/first visit to specialist psychiatric care by refugees’ country of birth, 2009–2012. N = 35.457

	Compulsory care		Inpatient care		Outpatient care	
	HR	95% CI	HR	95% CI	HR	95% CI
Country of birth						
Former Yugoslavia	1		1		1	
Other	1.58	0.86–2.90	1.41	1.13 −1.76	1.03	0.92–1.14
Horn of Africa	3.98	2.12–7.46	1.55	1.17–2.06	0.89	0.77–1.05
Iraq	1.52	0.78–2.96	1.03	0.80–1.34	1.05	0.93–1.19
Iran	3.07	1.52–6.19	1.84	1.37–2.47	1.64	1.43–1.88
Length of residence						
≤10 years	1		1		1	
≥11 years	0.56	0.27–1.13	1.24	0.92–1.67	1.22	1.05–1.41

HR, hazard ratio; CI, confidence intervals.

Models adjusted for unaccompanied/accompanied, length of residence, age, gender and domicile.

Other countries include South Asia, Latin America, other Africa and other Middle East.

### Education

Interaction analyses found that the use of psychiatric care services was similar in men and women. However, when we examined education, there were significant interactions between the level of education and migrant status in the use of psychiatric care services. The results from the analyses stratified by educational level (see Supplementary Table C) indicated that the use of psychiatric care increased with the level of education in the refugee population, while the opposite was true for the general Swedish population. In the population with secondary education, the risk of compulsory admission was particularly high among unaccompanied refugees, with an HR of 4.72 (3.06–7.29), and accompanied refugees, with an HR of 2.04 (1.51–2.73), than the general Swedish population. The opposite was true for those who had only received primary education.

## Discussion

This register study compared a national cohort of 35 457 young adult refugees, who settled in Sweden in their teens, and 1 255 782 peers from the general Swedish population. The results showed that refugees were more likely than the general population to be admitted to a psychiatric hospital for inpatient and compulsory care, but not for outpatient care. However, the refugees’ use of outpatient psychiatric care increased the longer that they lived in Sweden. Refugees born in the Horn of Africa and Iran were most likely to undergo compulsory admission to hospital care and to receive other forms of inpatient care.

We found a contrast between the very high rates of compulsory psychiatric care in certain ethnic groups, like those from the Horn of Africa and Iran, but similar rates of outpatient care compared with the general Swedish population. We may, therefore, speculate that there were barriers to accessing psychiatric care in the early or milder stages of psychiatric disorders for some refugee populations. These barriers refer to: the refugees’ knowledge about the healthcare system, their language skills, their low health literacy levels, cultural factors affecting their self-perceived needs and their use of healthcare. Another barrier was a lack of competence in transcultural psychiatry by Swedish staff (Uba, 1992; Zanchetta & Poureslami, 2006; Norredam *et al.* 2007; Brendler-Lindqvist *et al.* 2014). A UK study (Mann *et al.* 2014) found similar patterns of care for patients with psychosis among the Black population in inner-city London. Such patterns could be attributed to factors in the organisation of psychiatric care in Sweden, like insufficient resources in neighbourhoods where refugees cluster (Gaskin *et al.* 2009). The differences between the various refugee groups also point to socio-cultural factors within the refugee populations, which have an impact on their self-perceived needs for psychiatric care (Leclere *et al.* 1994; Lay *et al.* 2007; Norredam *et al.* 2010). These included fear of the stigma of mental illness and anticipated rejection (Corrigan, 2004), as well as different ways in which individuals coped with traumatic events (Alvidrez, 1999; Fenta *et al.* 2007).

To the best of our knowledge, this is the first Scandinavian study to report higher rates of compulsory psychiatric care for certain non-European refugee populations compared with the general population. However, similar findings have been repeatedly reported in non-white members of ethnic minorities in the UK from the 1980s onwards. These findings have been interpreted as the results of structural racism, embedded within factors such as social institutions and policies (Cope, 1989). In addition, it has been shown that Western psychiatrists perceive non-white patients as more dangerous than similar patients with white skin (Littlewood, 1992). There are also studies that indicate that psychosocial adversity and socio-economic disadvantages faced by ethnic minorities could play a legitimate role in explaining the high risks of mental health deterioration (Hollander *et al.* 2016), that could lead to compulsory referrals (Riecher *et al.* 1991; Nazroo, 1998).

This study showed that refugees who settled in Sweden as unaccompanied teenagers were more likely to be admitted to inpatient psychiatric care than teenage refugees who settled with their families. This was in line with the higher psychiatric morbidity reported in this vulnerable refugee group in recent epidemiological studies from Belgium, Sweden, the Netherlands and Norway (Bean *et al.* 2007*a*; Seglem *et al.* 2011; Eide & Hjern, 2013; Ramel *et al.* 2015). Our findings also showed that living in Sweden for a long time was associated with increasing risks of needing outpatient care. It is possible that this can be explained by refugees’ acculturation with Swedish values and the way that the general population seeks help with mental health issues. This view is in line with previous studies that have linked acculturation with mental health among the migrant population (Rogler *et al.* 1991; Norredam *et al.* 2010; Bhugra *et al.* 2011; Durbin *et al.* 2015).

It has been argued that education and can be important predictors of how people use healthcare services, but low levels of education and health literacy can act as barriers to accessing healthcare (Scheppers *et al.* 2006; Zanchetta & Poureslami, 2006). This suggests that education and health literacy in general can play a key role in increasing knowledge about the healthcare system, self-perceived need of healthcare, so that refugees can take full advantage of the care on offer (Eriksson-Sjöö *et al.* 2012).

In addition, one could also speculate that refugees with higher levels of education do not benefit from the same improvements in income and employment as the native Swedish population. An intriguing pattern emerged from our study and that was that all psychiatric care, including compulsory detention, demonstrated a reversed educational gradient in refugees compared with the general Swedish population. This finding warrants further studies that also include data about employment and income.

## Strengths, limitations and methodological issues

The major strength of our study was that it used national register data covering the entire population living in Sweden during the follow-up period. The dataset resulted from a combination of different registers and we used the unique personal number allocated to all Swedish citizens to link the socio-demographic characteristics of individuals with their use of psychiatric care services. A further strength lay in the fact that our data allowed us to identify accompanied and unaccompanied refugees living in Sweden as a result of the Geneva Convention, as well as refugees granted residence permits in Sweden on humanitarian grounds.

Besides its obvious strengths, this study also had some limitations. Firstly, our data did not provide information about the actual mental health status of the study population. As a result, we needed to be careful about drawing definitive conclusions about the reasons behind the patterns of use of psychiatric care services by refugees. Secondly, essential information about the mechanisms underlying the associations was lacking, such as information about cultural values, social support or different types of ethnic discrimination that could account for the different patterns in care-seeking behaviours. In addition, we could not estimate to what extent the variations in use of psychiatric care by country or origin reflected different care-seeking behaviours and barriers to care. The others category used in the country of birth variable contained important population heterogeneity that should be considered when interpreting the final results. Another noteworthy shortcoming of our study was related to the date of immigration and how long the refugees had lived in Sweden, as this was based on the date when a residence permit was granted, not the date when the person entered Sweden. Thus, it is possible that some refugees could have lived in Sweden for a longer period of time than estimated. In addition, the inclusion of substance abuse would have been a valuable addition to this study. However, because data on substance abuse are collected by a separate branch of the Swedish psychiatric care service, we decided to address this particular issue in a forthcoming study.

## Implications for research, policy and practice

The results from this study indicate that specialist outpatient psychiatric care was underused by refugees living in Sweden during the study period. Further studies are needed to identify the specific barriers that explain this pattern. This will help to inform the development of health policies that are more sensitive to the special geographical, linguistic and cultural needs of this vulnerable population.

## Availability of data and materials

According to Swedish data protection laws, administrative data is made available for specific research projects. Thus, we cannot share the data we used for this study with other researchers. We, however, are happy to answer any questions about the data used in this study and to share unpublished results.

## Supplementary material

For supplementary material accompanying this paper visit http://dx.doi.org/10.1017/S2045796016000445. click here to view supplementary material

## References

[ref1] Alvidrez J (1999). Ethnic variations in mental health attitudes and service use among low-income African American, Latina, and European American young women. Community Mental Health Journal 35, 515–530. 10863988 10.1023/a:1018759201290

[ref2] Bean T, Derluyn I, Eurelings-Bontekoe E, Broekaert E, Spinhoven P (2007 *a*). Comparing psychological distress, traumatic stress reactions, and experiences of unaccompanied refugee minors with experiences of adolescents accompanied by parents. Journal of Nervous and Mental Disease 195, 288–297. 17435478 10.1097/01.nmd.0000243751.49499.93

[ref3] Bean TM, Eurelings-Bontekoe E, Spinhoven P (2007 *b*). Course and predictors of mental health of unaccompanied refugee minors in the Netherlands: one year follow-up. Social Science and Medicine 64, 1204–1215. 17188787 10.1016/j.socscimed.2006.11.010

[ref4] Bhugra D, Gupta S, Bhui K, Craig T, Dogra N, Ingleby JD, Kirkbride J, Moussaoui D, Nazroo J, Qureshi A (2011). WPA guidance on mental health and mental health care in migrants. World Psychiatry 10, 2–10. 21379345 10.1002/j.2051-5545.2011.tb00002.x PMC3048516

[ref5] Brendler-Lindqvist M, Norredam M, Hjern A (2014). Duration of residence and psychotropic drug use in recently settled refugees in Sweden – a register-based study. International Journal for Equity in Health 13, 1–9. 25526935 10.1186/s12939-014-0122-2 PMC4297375

[ref6] Cope R (1989). The compulsory detention of Afro-Caribbeans under the Mental Health Act. Journal of Ethnic and Migration Studies 15, 343–356.

[ref7] Corrigan P (2004). How stigma interferes with mental health care. American Psychologist 59, 614. 15491256 10.1037/0003-066X.59.7.614

[ref8] Davidson N, Skull S, Chaney G, Frydenberg A, Isaacs D, Kelly P, Lampropoulos B, Raman S, Silove D, Buttery J (2004). Comprehensive health assessment for newly arrived refugee children in Australia. Journal of Paediatrics and Child Health 40, 562–568. 15367154 10.1111/j.1440-1754.2004.00465.x

[ref9] Derluyn I, Mels C, Broekaert E (2009). Mental health problems in separated refugee adolescents. Journal of Adolescent Health 44, 291–297. 19237116 10.1016/j.jadohealth.2008.07.016

[ref10] Durbin A, Moineddin R, Lin E, Steele LS, Glazier RH (2015). Mental health service use by recent immigrants from different world regions and by non-immigrants in Ontario, Canada: a cross-sectional study. BMC Health Services Research 15, 336. 26290068 10.1186/s12913-015-0995-9 PMC4546085

[ref11] Eide K, Hjern A (2013). Unaccompanied refugee children–vulnerability and agency. Acta Paediatrica 102, 666–668. 23560773 10.1111/apa.12258

[ref12] Eriksson-Sjöö T, Cederberg M, Östman M, Ekblad S (2012). Quality of life and health promotion intervention-a follow up study among newly-arrived Arabic-speaking refugees in Malmö, Sweden. International Journal of Migration, Health and Social Care 8, 112–126.

[ref13] Fazel M, Reed RV, Panter-Brick C, Stein A (2012). Mental health of displaced and refugee children resettled in high-income countries: risk and protective factors. The Lancet 379, 266–282. 10.1016/S0140-6736(11)60051-2 21835459

[ref14] Fenta H, Hyman I, Noh S (2007). Health service utilization by Ethiopian immigrants and refugees in Toronto. Journal of Immigrant and Minority Health 9, 349–357. 17380388 10.1007/s10903-007-9043-0

[ref15] Gaskin DJ, Price A, Brandon DT, LaVeist TA (2009). Segregation and disparities in health services use. Medical Care Research and Review 66, 578–589. 19460811 10.1177/1077558709336445 PMC3099538

[ref16] Hjern A (2004). Illicit drug abuse in second-generation immigrants: a register study in a national cohort of Swedish residents. Scandinavian Journal of Public Health 32, 40–46. 14757547 10.1080/14034940310001677

[ref17] Hjern A (2012). Migration and public health Health in Sweden: the National Public Health Report 2012. Chapter 13. Scandinavian Journal of Public Health 40, 255–267. 23238411 10.1177/1403494812459610

[ref18] Hjern A, Angel B (2000). Organized violence and mental health of refugee children in exile: a six-year follow-up. Acta Paediatrica 89, 722–727. 10914971 10.1080/080352500750044089

[ref19] Hjern A, Wicks S, Dalman C (2004). Social adversity contributes to high morbidity in psychoses in immigrants – a national cohort study in two generations of Swedish residents. Psychological Medicine 34, 1025–1033. 15554573 10.1017/s003329170300148x

[ref20] Hollander A-C, Dal H, Lewis G, Magnusson C, Kirkbride JB, Dalman C (2016). Refugee migration and risk of schizophrenia and other non-affective psychoses: cohort study of 1.3 million people in Sweden. BMJ 352, i1030. 26979256 10.1136/bmj.i1030 PMC4793153

[ref21] Howard M, Hodes M (2000). Psychopathology, adversity, and service utilization of young refugees. Journal of the American Academy of Child and Adolescent Psychiatry 39, 368–377. 10714058 10.1097/00004583-200003000-00020

[ref22] Jablonska B, Lindberg L, Lindblad F, Hjern A (2009). Ethnicity, socio-economic status and self-harm in Swedish youth: a national cohort study. Psychological Medicine 39, 87. 18366815 10.1017/S0033291708003176

[ref23] Kirmayer LJ, Narasiah L, Munoz M, Rashid M, Ryder AG, Guzder J, Hassan G, Rousseau C, Pottie K (2011). Common mental health problems in immigrants and refugees: general approach in primary care. Canadian Medical Association Journal 183, E959–E967. 20603342 10.1503/cmaj.090292 PMC3168672

[ref24] Lay B, Nordt C, Rössler W (2007). Mental hospital admission rates of immigrants in Switzerland. Social Psychiatry and Psychiatric Epidemiology 42, 229–236. 17450403 10.1007/s00127-007-0157-4

[ref25] Leclere FB, Jensen L, Biddlecom AE (1994). Health care utilization, family context, and adaptation among immigrants to the United States. Journal of Health and Social Behavior 370–384. 7844331

[ref26] Littlewood R (1992). Psychiatric diagnosis and racial bias: empirical and interpretative approaches. Social Science and Medicine 34, 141–149. 1738867 10.1016/0277-9536(92)90091-4

[ref27] Ludvigsson JF, Andersson E, Ekbom A, Feychting M, Kim J-L, Reuterwall C, Heurgren M, Olausson PO (2011). External review and validation of the Swedish national inpatient register. BMC Public Health 11, 450. 21658213 10.1186/1471-2458-11-450 PMC3142234

[ref28] Mann F, Fisher HL, Major B, Lawrence J, Tapfumaneyi A, Joyce J, Hinton MF, Johnson S (2014). Ethnic variations in compulsory detention and hospital admission for psychosis across four UK early intervention services. BMC Psychiatry 14, 256. 25214411 10.1186/s12888-014-0256-1 PMC4173060

[ref29] Migrationsverket (2015). Aktuellt om ensamkommande barn & ungdomar. Retrieved 16 May 2016 from http://www.migrationsverket.se/download/18.2d998ffc151ac387159be0/1451642643512/%C3%96versikt+statistik+2015.pdf.

[ref30] Montgomery E (2010). Trauma and resilience in young refugees: a 9-year follow-up study. Development and Psychopathology 22, 477–489. 20423554 10.1017/S0954579410000180

[ref31] Nazroo J (1998). Rethinking the relationship between ethnicity and mental health: the British Fourth National Survey of Ethnic Minorities. Social Psychiatry and Psychiatric Epidemiology 33, 145–148. 9567663 10.1007/s001270050036

[ref32] Norredam ML, Nielsen AS, Krasnik A (2007). Migrants’ access to healthcare. Danish Medical Bulletin 54, 48–49. 17349225

[ref33] Norredam M, Nielsen SS, Krasnik A (2010). Migrants’ utilization of somatic healthcare services in Europe – a systematic review. The European Journal of Public Health 20, 555–563. 20040522 10.1093/eurpub/ckp195

[ref34] Ramel B, Täljemark J, Lindgren A, Johansson BA (2015). Overrepresentation of unaccompanied refugee minors in inpatient psychiatric care. SpringerPlus 4, 131–131. 25825687 10.1186/s40064-015-0902-1 PMC4372620

[ref35] Riecher A, Rössler W, Löffler W, Fätkenheuer B (1991). Factors influencing compulsory admission of psychiatric patients. Psychological Medicine 21, 197–208. 2047496 10.1017/s0033291700014781

[ref36] Rogler LH, Cortes DE, Malgady RG (1991). Acculturation and mental health status among Hispanics: convergence and new directions for research. American Psychologist 46, 585. 1952420 10.1037//0003-066x.46.6.585

[ref37] Rosén M (2002). National health data registers: a Nordic heritage to public health. Scandinavian Journal of Public Health 30, 81–85. 12028856 10.1080/140349401753683444

[ref38] Scheppers E, Van Dongen E, Dekker J, Geertzen J, Dekker J (2006). Potential barriers to the use of health services among ethnic minorities: a review. Family Practice 23, 325–348. 16476700 10.1093/fampra/cmi113

[ref39] Seglem KB, Oppedal B, Raeder S (2011). Predictors of depressive symptoms among resettled unaccompanied refugee minors. Scandinavian Journal of Psychology 52, 457–464. 21895671 10.1111/j.1467-9450.2011.00883.x

[ref40] Uba L (1992). Cultural barriers to health care for southeast Asian refugees. Public Health Reports 107, 544. 1410235 PMC1403696

[ref41] Weitoft GR, Gullberg A, Hjern A, Rosén M (1999). Mortality statistics in immigrant research: method for adjusting underestimation of mortality. International Journal of Epidemiology 28, 756–763. 10480707 10.1093/ije/28.4.756

[ref42] ZanchettaMS, PoureslamiIM (2006). Health literacy within the reality of immigrants’ culture and language. Canadian Journal of Public Health/Revue Canadienne de Sante'e Publique S26–S30.16805158

