# Supplementary material for: The use of psychiatric services by young adults who came to Sweden as teenage refugees: a national cohort study
Source: Epidemiol Psychiatr Sci. 2016 Jun 29;26(5):526–34. doi: 10.1017/S2045796016000445 (PMC6999002; doi:10.1017/S2045796016000445)
Supplement: Supplementary file 1 [file S2045796016000445sup001.docx]

**Appendix**

**Table A. Rates of having at least one visit to specialist psychiatric services by country of birth**

| Country of birth |  | N |  | Compulsory care (%) | |  | Inpatient care (%) | |  | Outpatient care (%) | |
| --- | --- | --- | --- | --- | --- | --- | --- | --- | --- | --- | --- |
|  |  |  |  | *Unaccompanied* | *Accompanied* |  | *Unaccompanied* | *Accompanied* |  | *Unaccompanied* | *Accompanied* |
| Native Swedish |  | *1.255.782* |  | 0.2* | - |  | 1.3* | - |  | 6.5* | - |
| Former Yugoslavia |  | 10.477 |  | 0.1 | 0.2 |  | 1.4 | 1.5 |  | 7.8 | 7 |
| Other |  | 10.613 |  | 0 | 0.4 |  | 2.3 | 1.9 |  | 6.9 | 6.8 |
| Horn of Africa |  | 4.093 |  | 0.8 | 0.7 |  | 1.8 | 2.2 |  | 5.8 | 5.7 |
| Iraq |  | 7.549 |  | 0.3 | 0.3 |  | 1.2 | 1.5 |  | 6.9 | 6.6 |
| Iran |  | 2.725 |  | 1.4 | 0.5 |  | 3.2 | 2.3 |  | 11.2 | 11 |

Other countries include South Asia, Latin America, other Africa and other Middle East; *Native Swedish

**Table B. Cox regression models for first hospital admission/first visit to specialist psychiatric care**

|  | Compulsory care |  | Inpatient care |  | Outpatient care |
| --- | --- | --- | --- | --- | --- |
|  | HR 95% CI |  | HR 95% CI |  | HR 95% CI |
| *Study Population* |  |  |  |  |  |
| Native Swedish | 1 |  | 1 |  | 1 |
| Accompanied refugees | 1.89 1.53-2.34 |  | 1.37 1.25-1.50 |  | 1.10 1.05-1.16 |
| Unaccompanied refugees | 2.76 1.86-4.10 |  | 1.62 1.34-1.94 |  | 1.29 1.18-1.41 |

HR=Hazard ratio; CI= confidence intervals; models adjusted for age, gender and domicile

**Table C. Cox regression models for first hospital admission/first visit to specialist psychiatric care stratified by level of education**

|  | Compulsory care | |  | Inpatient care | |  | Outpatient care | |
| --- | --- | --- | --- | --- | --- | --- | --- | --- |
|  | HR 95% CI | HR 95% CI |  | HR 95% CI | HR 95% CI |  | HR 95% CI | HR 95% CI |
| *Study population* | *Primary* | *Secondary* |  | *Primary* | *Secondary* |  | *Primary* | *Secondary* |
| Native Swedish | 1 | 1 |  | 1 | 1 |  | 1 | 1 |
| Unaccompanied refugees | 0.31 0.11-0.84 | 4.72 3.06-7.29 |  | 0.74 0.57-0.97 | 1.48 1.14-1.92 |  | 0.57 0.49-0.66 | 1.34 1.19-1.51 |
| Accompanied refugees | 0.69 0.51-0.94 | 2.04 1.51-2.73 |  | 0.62 0.54-0.70 | 1.35 1.19-1.52 |  | 0.51 0.47-0.54 | 1.14 1.07-1.20 |

HR=Hazard ratio; CI= confidence intervals; model adjusted for age, gender and domicile
